# Improving Virological Monitoring of HDV Infection: A Proof-of-Concept Comparative Study of Bosphore and AltoStar^®^ Assays in Patients Treated with Bulevirtide

**DOI:** 10.3390/biomedicines13071564

**Published:** 2025-06-26

**Authors:** Verdiana Zulian, Chiara Taibi, Antonio Coppola, Angela Bibbò, Luigi Federici, Martina De Sanctis, Silvia Pauciullo, Gianpiero D’Offizi, Elisa Biliotti, Fiona McPhee, Anna Rosa Garbuglia

**Affiliations:** 1 Virology Laboratory, National Institute for Infectious Diseases Lazzaro Spallanzani IRCCS, 00149 Rome, Italy; antonio.coppola@inmi.it (A.C.); angela.bibbo@inmi.it (A.B.); luigi.federici@inmi.it (L.F.); desanctis.1635418@studenti.uniroma1.it (M.D.S.); silvia.pauciullo@inmi.it (S.P.); annarosa.garbuglia@inmi.it (A.R.G.); 2 Infectious Diseases and Hepatology Unit, National Institute for Infectious Diseases Lazzaro Spallanzani IRCCS, 00149 Rome, Italy; chiara.taibi@inmi.it (C.T.); gianpiero.doffizi@inmi.it (G.D.); elisa.biliotti@inmi.it (E.B.); 3 Independent Researcher, North Devon, Westward Ho! EX39 1GZ, UK; mcpheef@gmail.com

**Keywords:** HDV RNA, molecular diagnostics, HBV/HDV detection, bulevirtide, outcome, virological response

## Abstract

Hepatitis delta virus (HDV) infection is associated with severe hepatic complications and rapid progression towards liver cirrhosis and hepatocellular carcinoma. Accurate measurement of HDV RNA is critical for monitoring therapeutic responses, especially during treatment with novel therapies such as bulevirtide (BLV). This study compared the analytical performance of two HDV RNA quantification assays, Bosphore (Anatolia) and AltoStar^®^ (Altona), focusing on their sensitivity, specificity, and potential implications for clinical management. Sixty-one clinical samples from twenty-four patients, including fifteen HDV-infected patients receiving BLV treatment and nine controls, were tested using each assay. Of 30 samples identified as HDV-negative by the Bosphore assay, 17 (56.7%) were HDV-positive with AltoStar^®^, demonstrating the superior sensitivity (*p* < 0.0001) of the latter assay. Quantitative analyses revealed consistently higher viral load measurements with AltoStar^®^ compared to Bosphore, with a difference of 1.23 Log IU/mL and a moderate correlation (r^2^ = 0.7385) between assays. Each assay demonstrated a high specificity, with no false positives detected among control samples. However, our findings suggest that differences in assay sensitivity could impact the evaluation of virological response, highlighting the risk of false-negative results in chronically HDV-infected patients with low-level viremia. This emphasizes the need for careful assay selection to accurately monitor treatment outcomes.

## 1. Introduction

The hepatitis delta virus (HDV) is a defective RNA virus that requires the hepatitis B virus (HBV) surface antigen (HBsAg) for the assembly of its virions. Its genome, approximately 1.2 kb in length, contains a single open reading frame encoding the HDV antigen, which exists in two isoforms: the short (S-HDAg) and long (L-HDAg) isoforms [1]. Despite its relatively simple structure, HDV induces significant hepatic damage and is regarded as the most virulent among hepatitis viruses. During the acute phase of infection, it can result in fulminant hepatitis. Moreover, HDV superinfection is associated with progression to chronic hepatitis in approximately 80% of cases [2]. In the chronic phase of the disease, HDV induces rapid progression towards cirrhosis and increases the risk of developing hepatocellular carcinoma (HCC) [3]. It has been estimated that over 15 million HBsAg-positive individuals worldwide also test positive for anti-HDV antibodies [4]. In the Eastern Mediterranean region, 15–37% of individuals chronically infected with HBV are coinfected with HDV [5]. Until recently, off-label therapy with pegylated interferon was the only available therapy for HDV infection. However, its use is associated with significant side effects, leading to treatment termination in some patients [6]. In 2020, a promising new antiviral drug, bulevirtide (BLV), with a recommended dose of 2 mg once daily by subcutaneous injection, received marked approval by the European Medical Agency (EMA) for the treatment of chronic HDV infection [7]. BLV is a lipopeptide derived from the preS1 domain sequence of HBV genotype C (residues Gly2-Gly48) with a shortening of 11 additional N-terminal amino acids and one amino acid substitution at position 46 (Gln46Lys) [8,9]. Several studies have demonstrated that the first 75 residues of the preS1 domain are essential for HBV and HDV infectivity as they mediate binding to the human Na+-taurocholate co-transporting polypeptide (NTCP), a member of the solute carrier transporter family, involved in the enterohepatic circulation of bile salts [10,11]. BLV acts as an entry inhibitor by specifically binding to the NTCP receptor, thereby preventing the attachment and entry of both HBV and HDV into hepatocytes. The virological response to BLV monotherapy is defined as either undetectable HDV RNA or a two-log decrease in HDV RNA after 24 weeks (T24w) of therapy. However, for patients who discontinued therapy, a rebound in HDV RNA has been observed [12]. This highlights the need for identification of predictive factors to enhance a long-term response during BLV treatment. Furthermore, one of the major challenges in defining the virological response lies in the variability of assay sensitivity for the determination of HDV RNA and the heterogeneity of measurement units used for HDV RNA quantification, which sometimes complicate data comparisons across studies [13,14]. Assays with low sensitivity may fail to detect low-level viremia, leading to an underestimation of residual viral load, corresponding to a low copy number of HDV RNA. In a clinical scenario where BLV monotherapy is halted in patients who appear to achieve and maintain long-term undetectable viremia, the failure to detect minimal residual HDV RNA could significantly increase the risk of viral reactivation upon therapy withdrawal [12].

In this study, we compared HDV RNA levels obtained using the Bosphore kit (Anatolia, Turkey) and the AltoStar^®^ version 1.5 kit (Altona, Hamburg, Germany, GmHB) in patients receiving BLV treatment. The comparison focused primarily on samples from patients with undetectable or low HDV RNA levels (<100 copies/mL), as measured by the Bosphore assay, and on samples from patients with high HDV RNA levels, to assess concordance with the more sensitive AltoStar^®^ assay.

## 2. Patients and Methods

### 2.1. Study Population

This study included 61 clinical samples obtained from 24 patients. Fifteen patients were coinfected with HBV and HDV (positive for HBsAg, anti-HDV antibody, and HDV RNA at baseline) and treated with BLV (2 mg/day), whereas nine patients were in the control group. The control group was represented by patients who were admitted to the Hepatology Unit for acute hepatitis unrelated to HBV/HDV infection (*n* = 4), and patients with resolved HDV infection (*n* = 5), either by liver transplantation or spontaneous clearance of the virus. Patients with resolved infection had been HDV RNA negative for at least 10 years.

Multiple samples were collected from 15 patients treated with BLV at different time points during treatment (*n* = 52), while one sample per individual was included for each of the 9 control patients.

Baseline characteristics of the 15 HBV/HDV coinfected patients are presented in Table 1.

To evaluate and compare the sensitivity of the Bosphore and AltoStar^®^ HDV assays, we selected a series of clinical samples that were initially tested using the Bosphore assay and subsequently re-analyzed with AltoStar^®^. The final sample set comprised 52 samples from the 15 patients treated with BLV monotherapy: (1) 30 samples with HDV RNA reported as “not detected” (ND) by the Bosphore assay; (2) 6 samples with HDV RNA detected but below the lower limit of quantification (LLoQ); and (3) 16 samples with quantifiable HDV RNA levels (≥LLoQ). Additionally, 9 samples from individuals who were anti-HDV antibody-negative or had resolved HDV infection were included as negative controls to confirm assay specificity. All selected samples were retested using the AltoStar^®^ assay. Anti-HDV antibodies were assessed using the DIAPRO assay (DIAPRO, Milan, Italy).

This study was approved by the local Ethics Committee of Regione Lazio (approval number 97/2023). Written informed consent was obtained from all participants, and all procedures were performed in accordance with the Declaration of Helsinki.

### 2.2. HDV RNA Quantification

Sample HDV RNA levels were first quantified using the Bosphore assay (Anatolia, Turkey). This assay is labeled with Conformité Européenne and is an in vitro diagnostic (IVD) test. Serum/plasma (400 µL) was processed using the automated QIAsymphony system (Qiagen, Hilden, Germany, GmbH), and nucleic acids were eluted in 60 µL of AVE buffer (QIAGEN). Real-time PCR was performed using 10 µL of the resultant eluate on a Rotor-Gene instrument (QIAGEN), following the manufacturer’s RT-PCR protocol and master mix. The lower limit of detection (LoD) was 45 copies/mL (5.4 IU/mL), and the lower limit of quantification (LLoQ) was 100 copies/mL (12 IU/mL), with a dynamic range of quantification of 10^2^–10^8^ copies/mL. The conversion factor from copies to international units (IUs) was determined to be 0.12 [15], based on the WHO international standard for HDV RNA, (PEI 7657/12) with a concentration of 575,000 IU/mL.

The AltoStar^®^ HDV RT-PCR Kit, with a LoD of 1.12 IU/mL, a LLoQ of 20 IU/mL, and an upper limit of quantification corresponding to 1,000,000 IU/mL, is a research-use-only (RUO) version. A total of 500 µL of serum/plasma was processed using the AltoStar^®^ AM6 system (Altona, Hamburg, Germany, GmbH) and nucleic acids were eluted in 80 µL of elution buffer. Real-time AltoStar^®^ was performed using 45 µL of the resultant eluate on a CFX96™ Deep Well Dx System (Bio-Rad Laboratories, Hercules, CA, USA).

Both the Bosphore and AltoStar^®^ assays utilized undisclosed proprietary primers and probes specific for subsequences of the hepatitis delta antigen region.

### 2.3. Statistical Analysis

Qualitative variables were expressed as percentages, while continuous variables were described using medians and interquartile ranges (IQRs) or ranges when appropriate. HDV RNA levels were converted to Log IU/mL prior to performing statistical analyses. Categorical and continuous variables were compared using Fisher’s exact test, the Mann–Whitney U test, and the Wilcoxon test, as appropriate. The McNemar test was applied to assess the sensitivity of the Bosphore and AltoStar^®^ assays in detecting HDV RNA. A linear regression analysis was conducted to explore the relationship between the results obtained by both assays and to determine the correlation coefficient (r^2^). Additionally, a Bland–Altman plot was generated to assess agreement between the two assays and to determine the bias index [16].

A *p*-value less than 0.05 was considered significant. Statistical analyses were performed using the RStudio version 2024.09.0 software (RStudio, Boston, MA, USA) [17] and GraphPad Prism 9 software (GraphPad software, Inc., La Jolla, CA, USA).

## 3. Results

**Figure 1:**
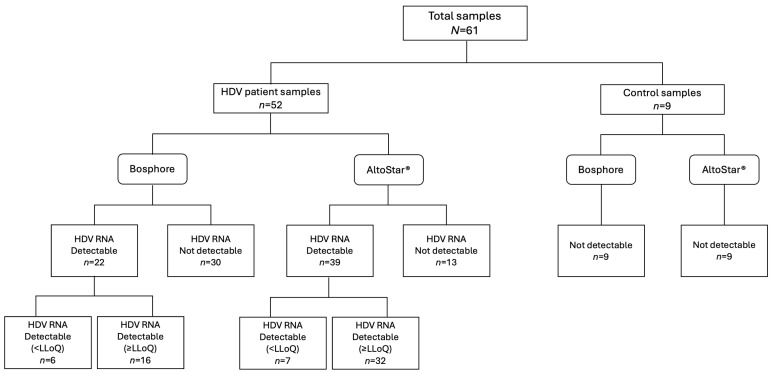
Distribution of HDV RNA quantification results for samples from patients treated with bulevirtide and control samples using the Bosphore and AltoStar^®^ assays, respectively. Kit-specified values for assay limits of detection (LoDs) and lower limits of quantification (LLoQs) were: Bosphore LoD < 45 cp/mL (<5.4 IU/mL) and LLoQ < 100 cp/mL (<12 IU/mL); AltoStar^®^ LoD < 1.12 IU/mL and LLoQ < 20.0 IU/mL.

A total of 61 clinical samples were analyzed using both the Bosphore and AltoStar^®^ assays. Fifty-two of these samples were obtained from 15 patients treated with BLV, and nine samples served as controls (Figure 1). All 15 patients treated with BLV were infected with HDV genotype 1 (12 with subtype 1e, 2 with subtype 1b, and 1 with an undefined subtype), as previously reported [18]. Among the 52 samples from patients receiving BLV, 16 were quantifiable with both the Bosphore and AltoStar^®^ assays. The median HDV RNA levels were 3.33 (min-max: 1.10–5.96) Log IU/mL with Bosphore and 5.01 (min-max: 3.55–6.74) Log IU/mL with AltoStar^®^ (*p* < 0.0001).

Qualitative analyses revealed that of thirty samples identified as “not detected” (ND) for HDV RNA using the Bosphore assay, only thirteen (43%) were confirmed as ND by the AltoStar^®^ assay. The remaining seventeen samples exhibited a median HDV RNA value of 1.50 Log IU/mL (IQR1-IQR3: 0.89–2.29 Log IU/mL), with values ranging from 0.55 to 3.08 Log IU/mL. Overall, HDV RNA detection was achieved in 57% more samples with the AltoStar^®^ assay than with the Bosphore assay, demonstrating a statistically significant increase in sensitivity [McNemar test, *p* < 0.0001 (95% CI: 19.94–45.44)]. Finally, six samples with HDV RNA levels <100 cp/mL (<12 IU/mL) in the Bosphore assay tested positive using the AltoStar^®^ assay, resulting in median values of 2.53 Log IU/mL (IQR1-IQR3: 2.44–2.91 Log IU/mL), ranging from 2.00 to 3.77 Log IU/mL. Furthermore, all samples quantifiable by Bosphore were also quantifiable by AltoStar^®^.

To investigate whether HDV RNA assay discrepancies were associated with host or viral factors, we compared HBsAg levels between patients with discordant results (i.e., HDV RNA not detected by Bosphore but detected by AltoStar^®^) and those with concordant results (i.e., HDV RNA not detected by both assays). No statistically significant difference in HBsAg levels was observed between the two groups (*p* = 0.77).

**Figure 2:**
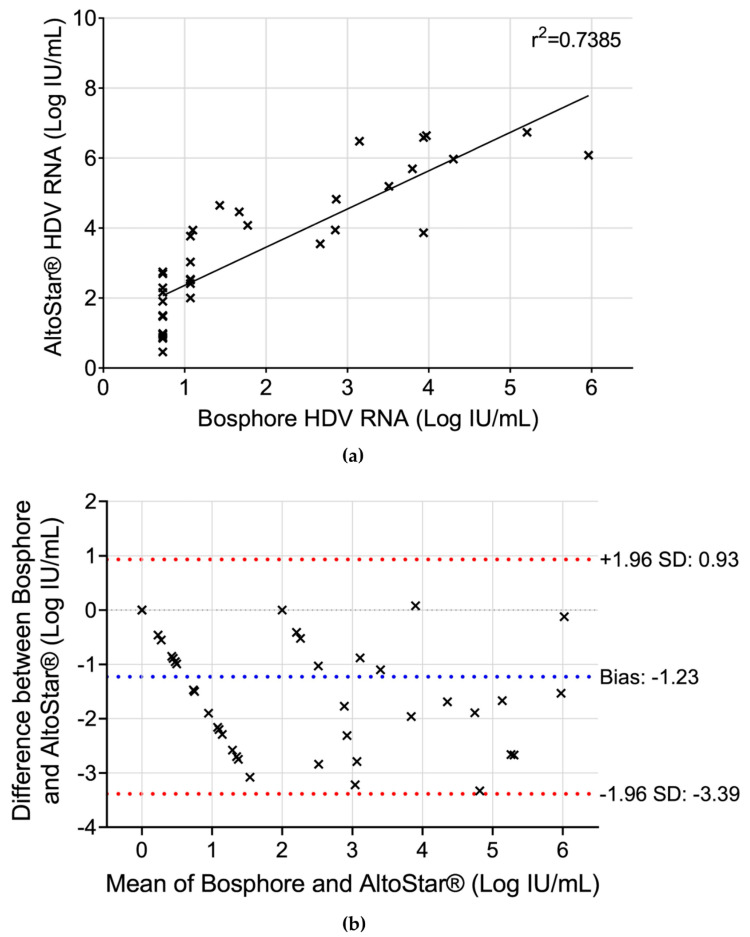
(**a**) Linear regression AltoStar^®^ versus Bosphore HDV RNA values. (**b**) Bland–Altman plot comparing HDV RNA values measured by AltoStar^®^ and Bosphore.

To explore the correlation between quantitative HDV RNA results obtained using the Bosphore and AltoStar^®^ kits, resultant Bosphore viral load values were converted from copies/mL into Log IU/mL, before conducting a linear regression analysis. The correlation coefficient (r^2^) between the two assays was 0.7385 (Figure 2a), indicating a moderate correlation. Variations between assays were primarily observed when HDV RNA values were assessed in IU/mL: the AltoStar^®^ assay consistently resulted in HDV RNA values approximately 1.23 Logs higher than those obtained with the Bosphore assay. Indeed, the Bland–Altman analysis revealed a bias index of −1.23 when comparing Bosphore to AltoStar^®^ (Figure 2b).

Overall, the greater sensitivity of the AltoStar^®^ assay may be attributed to utilizing a larger sample volume for HDV RNA detection. Specifically, AltoStar^®^ uses a sample volume corresponding to 281 µL, compared to 67 µL employed by Bosphore.

All control samples (*n* = 9) were negative for HDV, with both Bosphore and AltoStar^®^, indicating a high specificity for both assays.

In samples from seven patients treated with BLV, HDV RNA declines ≥2 Log were detected between visits when using the Bosphore assay (Table S1). However, this same decrease was not always observed with AltoStar^®^; in fact, for 2/7 (29%) patients, the HDV RNA decline reported with AltoStar^®^ was <2 Log between visits, specifically 0.55 and 1.57 Log IU/mL (*p* = 0.46).

For a subgroup of patients receiving BLV therapy (*n* = 10) and achieving an undetectable HDV RNA readout for the first time using the Bosphore assay (T1), viral load results were compared with those obtained with the AltoStar^®^ assay at T1 and after six months (T2) (Table 2). Notably, patients achieving ND or <10 IU/mL at T1, as measured by the AltoStar^®^ assay, maintained a ND readout with both assays at T2. Conversely, patients with HDV RNA >10 IU/mL at T1 with AltoStar^®^ continued to show persistent viremia at T2, with a median viral load of 2.29 Log IU/mL (range 0.95 to 3.03 Log IU/mL [*p* = 0.0048]). Therefore, low viremia (<10 IU/mL), as measured by AltoStar^®^, could be predictive of achieving undetectable HDV in subsequent months during BLV treatment.

## 4. Discussion

The main objective of this study was to compare the performance of the Bosphore and AltoStar^®^ assays for HDV RNA quantification and assess how discrepancies between the two assays could influence the evaluation of the virological response to BLV therapy. For this study, 61 clinical samples were included: 52 from 15 HDV-infected patients treated with BLV and 9 control samples from individuals who were negative for anti-delta antibodies or had a resolved HDV infection. Of the 52 patient samples tested using the Bosphore assay, 22 had detectable HDV RNA, including 6 samples with HDV RNA <LLoQ (<100 cp/mL, 12 IU/mL), while HDV RNA was not detected in 30. The retesting of these samples using the AltoStar^®^ assay identified 39 with detectable HDV RNA and 13 with no detectable HDV RNA. Therefore, this kit was able to detect HDV RNA in 13 (43.3%) samples that was not detected with Bosphore. HDV RNA was not detected in any control samples with either assay, suggesting that the Bosphore assay exhibited the same specificity as AltoStar^®^, but with a lower sensitivity. Detection of low viremia (<10 IU/mL) by the AltoStar^®^ assay appeared to be predictive of HDV RNA decreasing below the assay LoD after six months of treatment (Table 2 and Table S1). In contrast, the presence of viremia >10 IU/mL was associated with persistent viral replication six months later (*p* = 0.0048). Overall, higher HDV RNA values were recorded with the AltoStar^®^ assay than the Bosphore assay across all samples analyzed (median HDV RNA 5.01 vs. 3.33 Log IU/mL, bias = 1.23 Log IU/mL; *p* < 0.0001). Additionally, there was moderate agreement between the two methods, with a concordance correlation coefficient of 0.75, in line with findings from other comparative studies [19]. A similar level of concordance was recently reported by Sandmann et al. [20], who analyzed WHO HDV RNA standard dilutions and 28 clinical samples from HDV-infected patients using two versions of the Robogene HDV RNA quantification kit (version 2.0 and the updated version 3.0, CE-IVD pending). RNA extraction was performed manually or by automation (INSTANT virus RNA/DNA kit and INSTANT Virus RNA/DNA Kit FX2.0, ROBOSCREEN GmbH, Leipzig, Germany). While both versions showed comparable results for WHO standard dilutions, significant discrepancies were observed in clinical samples, with >1 Log IU/mL variability in 57% (16/28) of cases. In our study, the higher sensitivity observed with the AltoStar^®^ assay may be a consequence of technical differences between the two assays, particularly the greater sample volume used for amplification (281 µL for AltoStar^®^ vs. 67 µL for Bosphore), or a consequence of the AltoStar^®^ platform having an enhanced detection efficiency for HDV subtype 1e. Although both assays are validated for the detection of all eight HDV genotypes, no specific information is provided regarding their analytical sensitivity across subtypes. This gap is particularly relevant for genotype 1, which is the most prevalent worldwide [21]. In our study, all patients were infected with HDV genotype 1, including 12 out of 15 with subtype 1e, 2 out of 15 with subtype 1b, and 1 out 15 with an undefined subtype. Future studies should therefore include genotype-specific performance evaluations to identify potential limitations in detection sensitivity.

The extraction method is not expected to have significantly influenced results, since both QIAsymphony^®^ and AltoStar^®^ employ magnetic bead-based extraction procedures.

Considering the impact of the lower sensitivity of the Bosphore assay on the management of BLV therapy, this did not appear to influence the overall definition of the virological response. In fact, for 4 of the 10 patients with undetectable HDV RNA in the Bosphore assay that was detectable by AltoStar^®^, the classification of virological response remained unchanged, since a >2 Log decline in HDV RNA was recorded with each assay by week 24. However, in two patients, a >2 Log change between consecutive time points was recorded with the Bosphore assay that was not observed with the AltoStar^®^ assay. These findings suggest that the choice of HDV RNA quantification platform may affect the assessment of virological response, particularly when evaluating on-treatment HDV RNA kinetics, and underscore the need for multicenter studies involving three or more platforms to evaluate inter-assay variability and standardize HDV quantification across clinical settings.

Moreover, according to the recommendations of the European Association for the Study of the Liver (EASL) [22], all HBsAg-positive patients should be screened for HDV antibodies, and in case of positivity, HDV RNA testing should be performed as a reflex test. In our study, HDV RNA was not detected with the Bosphore assay in 6 out of 15 patients, while viral loads >100 IU/mL were recorded when tested with AltoStar^®^, highlighting the risk of false-negative results in chronically infected HDV patients with low-level viremia [23].

There were some limitations to our study. First, the sample size was relatively small. Second, paired baseline and week-24 samples for all HDV-infected study patients were not available for analysis. For these reasons, the results obtained cannot provide definitive conclusions regarding the level of sensitivity required for HDV RNA assays used to monitor anti-HDV therapy. Nevertheless, our findings suggest that higher assay sensitivity enables the detection of residual viremia, which may be relevant for evaluating potential treatment-stopping criteria and for improving the clinical management of chronic HDV infection. Finally, our findings highlight the need to establish clinically meaningful HDV RNA thresholds to guide BLV discontinuation and ensure sustained viral suppression.

## Figures and Tables

**Table 1:** Baseline characteristics of HBV/HDV coinfected patients receiving BLV therapy.

Parameter	*N* = 15
Age at recruitment, years	50 (47–62)
Male sex	4 (26.7)
Body-mass index	24 (23.0–26.5)
Cirrhosis	11 (73.3)
Previous interferon therapy	9 (60.0)
Concomitant NUC therapy	15 (100.0)
ALT, U/L	78.0 (46.5–100.0)
AST, U/L	70.0 (59.8–76.5)
Albumin, g/dL	4.2 (4.0–4.4)
Bile acids, μmol/L	9.1 (6.4–16.7)
Total bilirubin, mg/dL	0.9 (0.7–1.6)
Platelet count, ×10^3^/μL	90.0 (68.0–152.0)
HDV RNA, Log cp/mL	5.0 (3.4–5.7)
HBV DNA detectable *	0 (0.0)
HBsAg, Log IU/mL	4.0 (3.6–4.2)
HBeAg positive	0 (0.0)

* HBV DNA > 10 IU/mL. Parameter values are either expressed as number (%) or median (IQR1–IQR3). BLV, bulevirtide; IQR, interquartile range; NUC, nucleos(t)ide-analogue therapy; ALT, alanine aminotransferase; AST, aspartate aminotransferase; HDV, hepatitis delta virus; HBV, hepatitis B virus; HBsAg, hepatitis B surface antigen; HBeAg, hepatitis B e antigen.

**Table 2:** Assay comparisons of HDV RNA readouts at two different time points.

		AltoStar^®^ HDV RNA (IU/mL)		Bosphore HDV RNA (IU/mL)	
Patient ID	HDV Genotype	T1 *	T2 **	T1	T2
PT1	1e	ND ^a^	ND	ND	ND
PT2	1 unsubtyped	ND	ND	ND	ND
PT3	1e	30	9	ND	ND
PT4	1e	10	ND	ND	ND
PT5	1e	3	ND	ND	ND
PT6	1b	497	146	ND	ND
PT7	1e	559	32	ND	ND
PT8	1e	1190	1069	ND	<LLoQ ^b^
PT9	1e	158	345	ND	<LLoQ
PT10	1e	383	259	ND	<LLoQ

* T1, time point 1: first time point where HDV RNA was not detected with the Bosphore assay. ** T2, time point 2: six months after T1. ^a^ ND, not detected; ^b^ <LLoQ (<100 cp/mL; <12 IU/mL).

## Data Availability

The data presented in this study are available on request from the corresponding author.

## Supplementary Materials

The following supporting information can be downloaded at: https://www.mdpi.com/article/10.3390/biomedicines13071564/s1, Table S1: Summary of HDV RNA readouts for all clinical samples tested using the Bosphore and AltoStar^®^ assays.
