# Supplementary material for: Improving Virological Monitoring of HDV Infection: A Proof-of-Concept Comparative Study of Bosphore and AltoStar® Assays in Patients Treated with Bulevirtide
Source: Biomedicines. 2025 Jun 26;13(7):1564. doi: 10.3390/biomedicines13071564 (PMC12292241; doi:10.3390/biomedicines13071564)
Supplement: Supplementary file 1 [file biomedicines-13-01564-s001.zip › biomedicines-3592369-supplementary.pdf]

Table S1: Summary of HDV RNA readouts for all clinical samples tested using the Bosphore and AltoStar® assays

Patient/Control ID	HDV Genotype	Sample	Bosphore HDV RNA (IU/mL)	AltoStar® HDV RNA (IU/mL)
PT1	1e	PT1_1 (T1)	ND[a]	ND
		PT1_2	ND	ND
		PT1_3 (T2)	ND	ND
		PT1_4	ND	ND
PT2	1 unsubtyped	PT2_1 (T1)	ND	ND
		PT2_2	ND	ND
		PT2_3 (T2)	ND	ND
PT3	1e	PT3_1 (T1)	ND	29.76
		PT3_2	ND	7.04
		PT3_3 (T2)	ND	8.96
		PT3-4	ND	7.68
		PT3_5	ND	7.68
		PT3_6	ND	3.52
PT4	1e	PT4_1*	8614.80	7320.48
		PT4_2	12.60	8700.8
		PT4_3*	ND	195.36
		PT4_4 (T1)	ND	9.76
		PT4_5	ND	<LoD[b]
		PT4_6 (T2)	ND	ND
PT5	1e	PT5_1 (T1)	ND	2.88
		PT5_2	ND	<LoD
		PT5_3 (T2)	ND	ND
		PT5_4	ND	ND
		PT5_5	ND	ND
PT6	1b	PT6_1*	8600.40	3869880.32
		PT6_2*	46.80	29033.76
		PT6_3 (T1)	ND	497.28
		PT6_4 (T2)	ND	145.76
PT7	1e	PT7_1 (T1)	ND	558.88
		PT7_2	ND	80.32
		PT7_3 (T2)	ND	31.84
PT8	1e	PT8_1 (T1)	ND	1189.92
		PT8_2 (T2)	<LLoQ[c]	1069.44

PT9	1e	PT9_1 (T1)	ND	157.92
		PT9_2 (T2)	<LLoQ	345.23
PT10	1e	PT10_1 (T1)	ND	383.36
		PT10_2 (T2)	<LLoQ	258.56
		PT10_3	<LLoQ	329.60
PT11	1e	PT11_1*	20068.20	934821.92
		PT11_2*	198.36	3524.00
PT12	1e	PT12_1*	918379.80	1211800.32
		PT12_2	6273.00	493672.16
		PT12_3*	709.20	8836.80
PT13	1e	PT13_1*	3227.40	156789.92
		PT13_2	27.00	44643.04
		PT13_3*	<LLoQ	5861.92
PT14	1b	PT14_1*	9338.40	4389244.80
		PT14_2*	59.40	11944.48
PT15	1e	PT15_1*	160741.80	5456419.68
		PT15_2	1405.80	3034111.20
		PT15_3	727.20	66600.48
		PT15_4*	<LLoQ	100.64
Control 1		C1	ND	ND
Control 2		C2	ND	ND
Control 3		C3	ND	ND
Control 4		C4	ND	ND
Control 5		C5	ND	ND
Control 6		C6	ND	ND
Control 7		C7	ND	ND
Control 8		C8	ND	ND
Control 9		C9	ND	ND

Table legend: PT, patient treated with bulevirtide; T1, first sample timepoint where HDV RNA was not detected with the Bosphore assay; T2, six months after T1; *, samples used to compare the outcome for patients with an HDV RNA decline ≥2 Log; ND[a], HDV RNA not detected; <LoD[b], limit of detection AltoStar® assay (<1.12 IU/mL); <LLoQ[c], lower limit of quantification Bosphore assay (<100 copies/mL, <12 IU/mL).
